# Plastome phylogenomics of *Cephalotaxus* (Cephalotaxaceae) and allied genera

**DOI:** 10.1093/aob/mcaa201

**Published:** 2020-11-30

**Authors:** Yunheng Ji, Changkun Liu, Jacob B Landis, Min Deng, Jiahui Chen

**Affiliations:** 1 CAS Key Laboratory for Plant Diversity and Biogeography of East Asia, Kunming Institute of Botany, Chinese Academy of Sciences, Kunming, Yunnan, China; 2 Yunnan Key Laboratory for Integrative Conservation of Plant Species with Extremely Small Population, Kunming Institute of Botany, Chinese Academy of Sciences, Kunming, Yunnan, China; 3 School of Integrative Plant Science, Section of Plant Biology and the L. H. Bailey Hortorium, Cornell University, Ithaca, NY, USA; 4 School of Ecology and Environmental Science, Yunnan University, Kunming, Yunnan, China

**Keywords:** Gymnosperm, Cephalotaxaceae, Taxaceae, phylogeny, molecular dating, taxonomic delineation, recent speciation

## Abstract

**Background and Aims:**

*Cephalotaxus* is a paleo-endemic genus in East Asia that consists of about 7–9 conifer species. Despite its great economic and ecological importance, the relationships between *Cephalotaxus* and related genera, as well as the interspecific relationships within *Cephalotaxus*, have long been controversial, resulting in contrasting taxonomic proposals in delimitation of Cephalotaxaceae and Taxaceae. Based on plastome data, this study aims to reconstruct a robust phylogeny to infer the systematic placement and the evolutionary history of *Cephalotaxus.*

**Methods:**

A total of 11 plastomes, representing all species currently recognized in *Cephalotaxus* and two *Torreya* species, were sequenced and assembled. Combining these with previously published plastomes, we reconstructed a phylogeny of Cephalotaxaceae and Taxaceae with nearly full taxonomic sampling. Under a phylogenetic framework and molecular dating, the diversification history of *Cephalotaxus* and allied genera was explored.

**Key Results:**

Phylogenetic analyses of 81 plastid protein-coding genes recovered robust relationships between *Cephalotaxus* and related genera, as well as providing a well-supported resolution of interspecific relationships within *Cephalotaxus*, *Taxus*, *Torreya* and *Amentotaxus*. Divergence time estimation indicated that most extant species of these genera are relatively young, although fossil and other molecular evidence consistently show that these genera are ancient plant lineages.

**Conclusions:**

Our results justify the taxonomic proposal that recognizes Cephalotaxaceae as a monotypic family, and contribute to a clear-cut delineation between Cephalotaxaceae and Taxaceae. Given that extant species of *Cephalotaxus* are derived from recent divergence events associated with the establishment of monsoonal climates in East Asia and Pleistocene climatic fluctuations, they are not evolutionary relics.

## INTRODUCTION

The economically important genus *Cephalotaxus* Siebold & Zuccarini ex Endlicher consists of about 7–9 evergreen coniferous species occurring in Burma, China, India, Japan, Korea, Laos, Malaysia, Thailand and Vietnam (Fu, 1984; Fu *et al.*, 1999; Farjon, 2010; Lang *et al.*, 2013; Yang, 2017; J. W. Zhang *et al.*, 2019). According to fossil records, the earliest *Cephalotaxus* species was present in Europe by the middle Eocene (Manchester *et al.*, 2009) and expanded its distribution range into East Asia (north-eastern China) by the late Eocene (He and Tao, 1997). During the Miocene and Pliocene, *Cephalotaxus* was widespread across the Northern Hemisphere in Eurasia and North America (Miki, 1958; Givulescu, 1973; Mai and Walther, 1978; Wilde 1989; He and Tao, 1997; Kvaček and Walther, 1998; Meller, 1998; Kvaček and Rember, 2000; Walther and Kvaček, 2007). Therefore, the extant members of the genus have a relatively restricted distribution possibly resulting from range contraction and southward migration driven by decreasing global temperatures of the Neogene and Pleistocene glaciation events (Axelrod, 1959; Qian and Ricklefs, 2000; Manchester *et al.*, 2009). Because of its antiquity, *Cephalotaxus* is an excellent representative of relict plant lineages in East Asia (Manchester *et al.*, 2009), and extant species within this genus are hypothesized to be evolutionary relicts (Fu, 1984; Wu *et al.*, 2005).

*Cephalotaxus* is distinctive in having two-ovulate bracts in the seed cones (Fu *et al.*, 1999); vegetative characters including general folia morphology and growth habit closely resemble those of *Amentotaxus* Pilger, *Austrotaxus* Compton, *Pseudotaxus* W. C. Cheng, *Taxus* Linn. and *Torreya* Arnott. Although the monophyly of *Cephalotaxus* has been robustly supported by both morphological (Ghimire and Heo, 2014) and molecular evidence (Cheng *et al.*, 2000; Hao *et al.*, 2008; Gao *et al.*, 2015), its taxonomic affinities remain largely disputed. Historically, *Cephalotaxus* was placed into either Taxaceae (Eichler, 1889; Van Tieghem, 1891; Pilger, 1903; Takhtajan, 1953; Hart, 1987; Price, 1990; Stefanoviac *et al*, 1998; Christenhusz, 2011) or Cephalotaxaceae (Neger, 1907; Florin, 1948, 1954; Singh, 1961). Due to contradicting taxonomic proposals inferred from different evidence, it remains unresolved whether Cephalotaxaceae should be recognized as a separate family or merged back within Taxaceae. For instance, cladistic analysis based on morphological characters (Ghimire and Heo, 2014) as well as phylogenetic inference utilizing 18S ribosomal DNA sequences (Chaw *et al.*, 1997) and plastid *rbcL* and *matK* regions (Quinn *et al.*, 2002) suggest that Cephalotaxaceae should be merged into Taxaceae. However, phylogenetic analyses using a combination of plastid *matK* and nuclear ribosomal internal transcribed spacer (ITS) regions (Cheng *et al.*, 2000), plastid *matK*, *rbcL*, *trnL–trnF*, *psbA–trnH* and nuclear ITS (Hao *et al.*, 2008), nuclear *LFY* and *NLY* (Lu *et al.*, 2014), and transcriptomic data (Majeed *et al.*, 2018; Ran *et al.*, 2018) support the sister relationship between Cephalotaxaceae and Taxaceae. Nevertheless, a clear-cut delineation between the two families remains unresolved due to the ambiguous position of *Amentotaxus* and *Torreya*. Although the analyses by Ran *et al.* (2018) and X. Zhang *et al.* (2019) showed that these two genera are closer to Taxaceae, Cheng *et al.* (2000), Hao *et al.* (2008), Lu *et al.* (2014) and Majeed *et al.* (2018) proposed to include them within Cephalotaxaceae. These conflicts necessitate that the relationships between *Cephalotaxus* and its relatives require further investigation.

Additionally, the traditional classification of *Cephalotaxus* is constructed primarily based on folia morphologies, including leaf size, shape of leaves and leaf base, density and arrangement of leaves, etc. (Fu, 1984; Fu *et al.*, 1999; Farjon, 2010; Lang *et al.*, 2013; Yang, 2017; J. W. Zhang *et al.*, 2019). These characters are highly divergent, and are usually overlapping between species (Tripp, 1995), making morphology-based taxonomy particularly perplexing and challenging (J. W. Zhang *et al.*, 2019). Since the establishment of the genus by Endlicher (1847), *Cephalotaxus* has been the subject of several taxonomic revisions (Fu *et al.*, 1999; Farjon, 2010; Lang *et al.*, 2013; J. W. Zhang *et al.*, 2019). Yet, credible species delineation and unambiguous interspecific relationships within the genus remain unresolved. Given their great economic and ecological importance (Fu, 1984; Lang *et al.*, 2013), resolving the aforementioned problems will help the utilization and conservation of extant *Cephalotaxus* species, and deepen our understanding of the evolutionary history of gymnosperms.

Phylogenetic reconstruction based on limited sequence variation often results in poor resolution and low support in relationships, particularly at lower taxonomic levels (Rokas and Carroll, 2005; Whitfield and Lockhart, 2007; Philippe *et al.*, 2011). In contrast to Sanger sequencing, next-generation sequencing techniques, which are capable of generating orders of magnitude more data, have offered new approaches to resolving historical problems in plant phylogenetics (e.g. Parks *et al.*, 2009; Barrett *et al.*, 2014; Stull *et al.*, 2015; Zhang *et al.*, 2017; Carlsen *et al.*, 2018; Dong *et al.*, 2018; Li *et al.*, 2019). Plastid genome (plastome) DNA sequences, which harbour a large number of evolutionarily informative variation suitable for phylogenetic analysis, have been widely employed to reconstruct robust evolutionary relationships in phylogenetically challenging plant taxa (e.g. Simpson *et al.*, 2017; Uribe-Convers *et al.*, 2017; Heckenhauer *et al.*, 2019; Ji *et al.*, 2019*a*).

Here, we sequenced and assembled whole plastomes of nine species of *Cephalotaxus* and two species of *Torreya* using a low coverage genome sequencing approach. Based on plastome phylogenomic analyses and fossil-calibrated molecular dating, we aim to (1) elucidate the relationships between *Cephalotaxus* and related genera; (2) investigate interspecific relationships within *Cephalotaxus*; and (3) infer the history of species diversification for *Cephalotaxus*.

## MATERIALS AND METHODS

### Plant materials, shotgun sequencing, plastome assembly and comparison

According to the most recent taxonomic revisions on *Cephalotaxus* (Lang *et al.*, 2013; J. W. Zhang *et al.*, 2019), our taxon sampling covered all seven species (*Cephalotaxus alpina*, *C. fortunei*, *C. griffithii*, *C. hainanensis*, *C. harringtonii*, *C. nana* and *C. oliveri*) recognized in the genus. We also sampled *C. mannii* and *C. sinensis* whose taxonomic status as a recognized species differ according to different authors (Fu *et al.*, 1999; Farjon, 2010; Lang *et al.*, 2013; J. W. Zhang *et al.*, 2019), to investigate their relationships to their congeneric species. Voucher specimens (Table 1) were deposited at the herbarium of Kunming institute of Botany, Chinese Academy of Sciences (KUN).

**Table 1.** Taxa newly sequenced in this study with source, voucher and GenBank accession numbers

Taxa	Locality	Voucher	GenBank accession
*Cephalotaxus alpina*	Zixishan Mountain, Chuxiong, Yunnan, China	Y. Ji and C. Tao 012	MT555079
*C. fortunei*	Jinfoshan Mountain, Nanchuan, Chongqing, China	Y. Ji and C. Liu 084	MT555080
*C. griffithii*	Dulongjiang, Gongshan, Yunnan, China	GLGS Exp. 38714	MT555081
*C. hainanensis*	Jianfengling, Ledong, Hainan, China	T. Su *s. n.*	MT555082
*C. harringtonii*	Cultivated in the Botanical Garden of Kunming Institute of Botany	Y. Ji and X. Gong 002	MT555083
*C. mannii*	Cultivated in the Botanical Garden of Kunming Institute of Botany	Y. Ji and X. Gong 003	MT555084
*C. nana*	Fengxi, Zhuxi, Hubei, China	Y. Ji and C. Liu 095	MT555085
*C. oliveri*	Cultivated in the Botanical Garden of Kunming Institute of Botany	Y. Ji and X. Gong 001	MT555086
*C. sinensis*	Dengchigou, Baoxing, Sichuan, China	Y. Ji, W. Chen and T. Su 180	MT555087
*Torreya fargesii* var. *yunnanensis*	Cultivated in the Botanical Garden of Kunming Institute of Botany	Y. Ji and X. Gong 005	MT555088
*T. jackii*	Cultivated in the Botanical Garden of Kunming Institute of Botany	Y. Ji and X. Gong 004	MT555089

Total genomic DNA for each species was isolated from approx. 20 mg of silica gel-dried leaf tissues using the CTAB (cetyltrimethylammonium bromide) method (Doyle and Doyle, 1987). Purified genomic DNA (approx. 5 µg) was used to construct shotgun libraries with a TruSeq DNA Sample Prep Kit (Illumina, Inc., USA) following the manufacturer’s instructions. Paired-end sequencing was conducted on the Illumina HiSeq 2500 platform. Shotgun reads were subjected to the NGS QC Toolkit (Patel and Jain, 2012) to remove adaptors and low-quality reads with default parameters. Based on the remaining high-quality Illumina reads, *de novo* plastome assembly was performed using NOVOPlasty v2.7.0 (Dierckxsens *et al.*, 2017) with a *k*-mer of 31, and using the large subunit of the Rubisco gene (*rbcL*) of *C. oliveri* (AF456387) as the seed for iterative extension of contigs to recover the complete plastome of each species.

The newly generated plastomes were annotated with the Dual Organellar Genome Annotator database (Wyman *et al.*, 2004); the annotation of protein-coding genes was confirmed with a BLAST search against the NCBI protein database. Genes putatively annotated as tRNA were further verified by tRNAscan-SE 1.21 (Schattner *et al.*, 2005) with default parameters. To identify plastome arrangement events in *Cephalotaxus*, the newly generated plastomes were progressively aligned with those of four species representing closely related genera, *Amentotaxus*, *Torreya*, *Pseudotaxus* and *Taxus*, using the multiple genome alignment program Mauve 2.3.1 (Darling *et al.*, 2010). Moreover, all *Cephalotaxus* plastomes were aligned pairwise using the program mVISTA (Mayor *et al.*, 2000) under the LAGAN mode to investigate sequence divergence.

### Phylogenetic analyses

In addition to the nine *Cephalotaxus* plastomes newly generated in this study, a previously sequenced plastome of *C. wilsoniana*, which was treated as a conspecific variety (Fu *et al.*, 1999; Farjon, 2010) or a synonym of *C. harringtonii* (Lang *et al.*, 2013; J. W. Zhang *et al.*, 2019), was included in phylogenetic analyses. Moreover, six Cupressaceae plastomes, two *Amentotaxus* plastomes, eight *Torreya* plastomes, one *Pseudotaxus* plastome and 14 *Taxus* plastomes available in GenBank were incorporated into the dataset (Supplementary data Table S1). According to the backbone relationships of gymnosperms (Christenhusz *et al.*, 2011; Ran *et al.*, 2018), *Agathis danunara* and *Podocarpus totara* were selected as outgroups. Eighty-one plastid protein-coding genes commonly shared (Supplementary data Table S2) were aligned with MAFFT v7.4 (Katoh *et al.*, 2013). The best-fitting partition scheme was selected using PartitionFinder v2.1.1 (Lanfear *et al.*, 2017) with the ‘greedy’ search algorithm.

Both maximum likelihood (ML) and Bayesian inference (BI) approaches were used for phylogenetic inference. The ML analyses were conducted with RAxML-HPC BlackBox v8.1.24 (Stamatakis, 2006) with the most suitable model (GTR + G + I) for sequence substitution selected using Modeltest v3.7 (Posada and Crandall, 1998) with the Akaike information criterion (Posada and Buckley, 2004). Ten independent ML searches were performed with 1000 standard bootstrapping replicates. The BI analyses were performed using MRBAYES v 3.1.2 (Ronquist and Huelsenbeck, 2003). Markov chain Monte Carlo (MCMC) runs were initiated with a random tree for 1 million generations, with trees sampled every 100 generations. Trees from the first 250 000 generations were discarded as burn-in. The posterior probability (PP) values were computed based on the remaining trees.

### Divergence time estimation

The program BEAST v. 2.4.7 (Bouckaert *et al.*, 2014) was employed to estimate divergence time. We incorporated three fossils to calibrate the molecular clock. The crown age of the lineage including Cupressaceae, Cephalotaxaceae and Taxaceae was set to 197 million years ago (Mya) (Florin, 1958, 1963). The most recent common ancestor of Cephalotaxaceae and Taxaceae was set to a minimum age of 163.5 Mya (Florin, 1963). The stem age of *Taxus* was set to a minimum age of 100 Mya (Xu *et al.*, 2015). We used the uncorrelated log-normal relaxed molecular clock approach, which allows uncertainty in the age of calibrations to be represented as prior distributions rather than as strict calibration/fixed points. The BEAST analyses were conducted under the sequence substitution model (GTR + G + I) selected by Modeltest with a Yule tree prior. Two independent replications of MCMC simulations were run, sampling every 1000 generations. The stationarity of the chains and convergence of MCMC simulations were monitored by Tracer v1.7.1 (Rambaut *et al.*, 2018). After stationarity was obtained, a maximum credibility tree was constructed with TreeAnotator v1.10 (http://beast.bio.ed.ac.uk/TreeAnnotator), with the first 5000 trees discarded as burn-in.

## RESULTS

### *Features of* Cephalotaxus *plastomes*

**Fig. 1.**
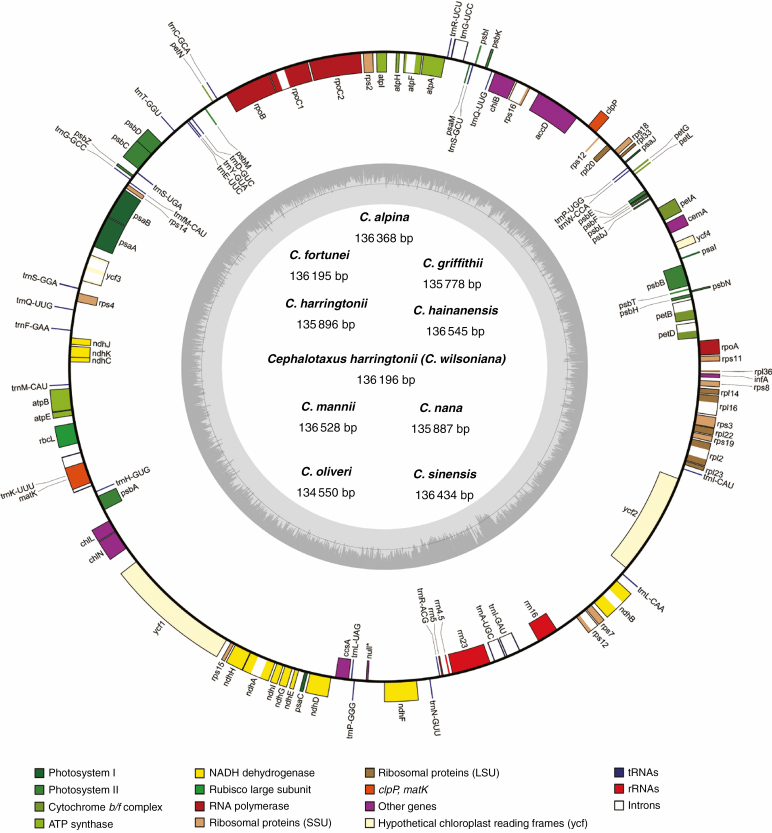
Map of *Cephalotaxus* plastomes. Genes shown outside the circle are transcribed clockwise, and those inside are transcribed counterclockwise. The dark grey area in the inner circle indicates the CG content of the plastome.

Shallow-depth genome sequencing produced approx. 30.1–31.5 million paired-end clean reads per sample. Of those, 4.16 × 10^5^–1.00 × 10^6^ were mapped to the assembled plastomes, with the average sequencing coverage ranging from 464× to 1117× (Supplementary data Table S3). The *de novo* assemblies generated nine circular *Cephalotaxus* plastomes, with the sizes of the complete plastome, coding regions and non-coding regions varying from 134 550 to 136 545 bp, 82 928 to 83 985 bp and 51 622 to 52 707 bp, respectively (Supplementary data Table S4). All *Cephalotaxus* species possessed a similar GC content not only in the whole plastomes assembly (35.1–35.2 %), but also in coding (37.3–37.5 %) and non-coding (31.%–31.6 %) regions (Supplementary data Table S4). All *Cephalotaxus* plastomes identically encoded 113 unique genes, comprising four rRNA genes, 27 tRNA genes and 82 protein-coding genes (Supplementary data Table S5). In addition, due to the loss of the typical inverted repeat region, it is difficult to define the boundary of large single-copy and small single-copy regions in *Cephalotaxus* plastomes (Fig. 1).

**Fig. 2.**
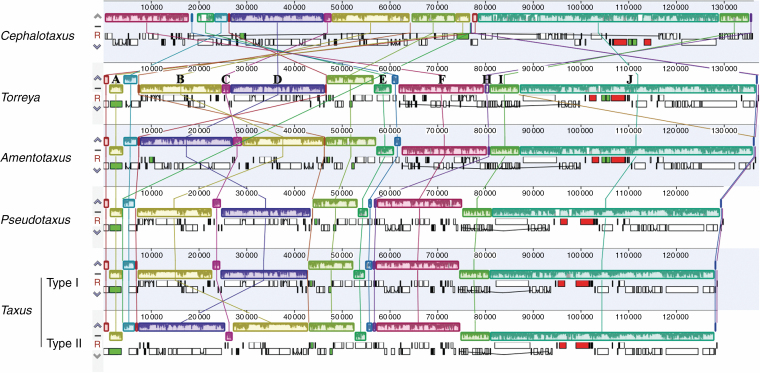
Multiple alignment resulted from Mauve showing genic arrangements detected in plastomes of *Cephalotaxus* and allied genera. Colour bars indicate syntenic blocks, and connecting lines indicate correspondence of blocks across plastomes.

**Fig. 3.**
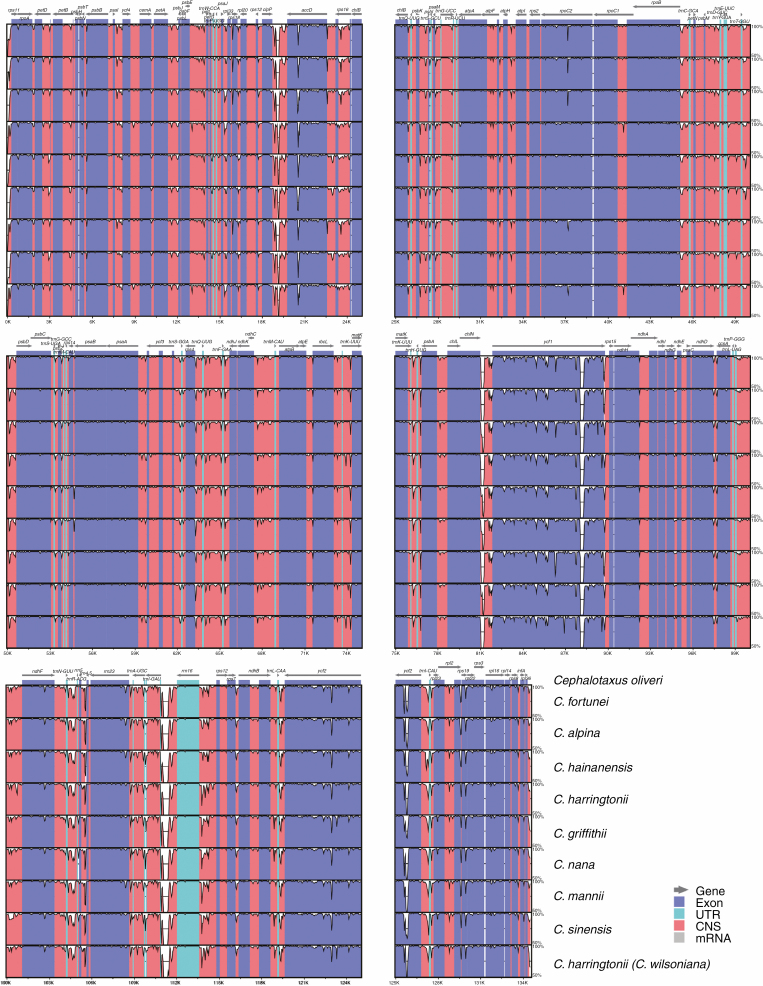
Alignment of *Cephalotaxus* plastomes using mVISTA, showing the percentages of sequence identity (*y*-axis).

Compared with *Amentotaxus*, *Pseudotaxus*, *Taxus* and *Torreya*, massive genic rearrangements were identified in *Cephalotaxus* by the progressive Mauve alignment of plastomes (Fig. 2). These variations led to *Cephalotaxus* plastomes being drastically different in gene arrangement from other Taxaceae genera. Nevertheless, *Cephalotaxus* plastomes exhibit high levels of sequence similarity at the species level. Plastome-wide mVISTA analyses (Fig. 3) detected a total of 2782 variations within the 139 245 alignment positions, representing 2 % variation in proportion among *Cephalotaxus* plastomes.

### Phylogenetic reconstruction

**Fig. 4.**
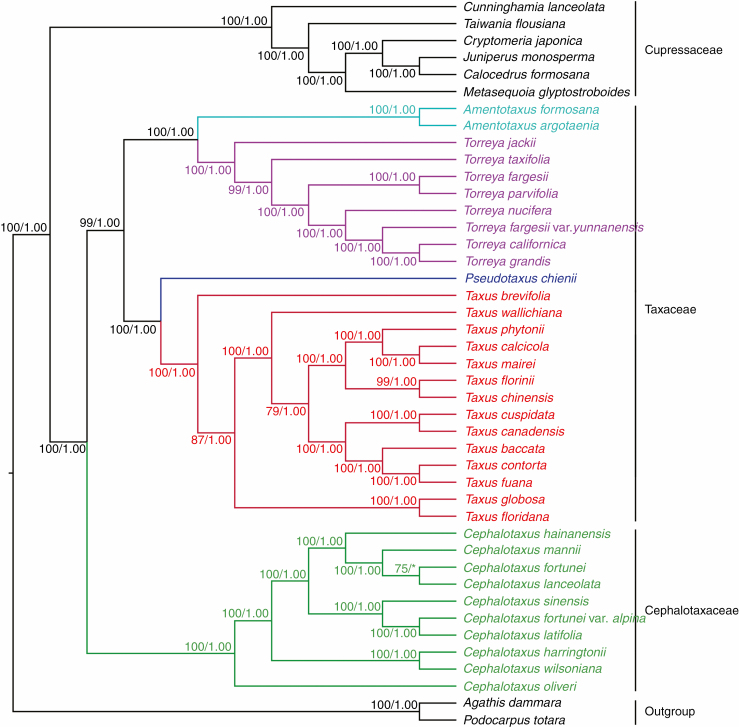
Phylogenetic relationships between *Cephalotaxus* and allied genera reconstructed by analyses of 81 plastid protein-coding genes using maximum likelihood (ML) and Bayesian inference (BI) methods. Numbers above branches indicated ML bootstrap percentage (BS) and BI posterior probability (PP).

The ML and BI analyses generated highly congruent tree topologies. The monophyly of *Cephalotaxus* (Cephalotaxaceae) was supported with 100 % bootstrap (BS) and 1.0 PP in ML and BI phylogenies. Its sister clade (Taxaceae) consisting of *Taxus*, *Pseudotaxus*, *Torreya* and *Amentotaxus* was fully supported (BS = 100 %, PP = 1.0) as well. Our plastome phylogenomic analyses recovered well-supported relationships among the Taxaceae genera: the four genera were grouped into two well-supported branches in the phylogenetic tree (BS = 100 %, PP = 1.0), in which the sister relationship between *Taxus* and *Pseudotaxus*, as well as between *Torreya* and *Amentotaxus*, received strong support (BS = 100 %, PP = 1.0). Within *Cephalotaxus*, *C. oliveri* was the earliest diverging species and sister to the clade consisting of the remaining species. *Cephalotaxus harringtonii* was sister to the clade including the other species, with this clade further containing two sister clades, one including four species (*C. hainanensis*, *C. mannii*, *C. fortunei* and *C. griffithii*), and the other including three species (*C. sinensis*, *C. alpina* and *C. nana*). Overall, our plastome-based phylogeny satisfactorily resolved the infrageneric relationships of *Cephalotaxus*: except for the *C. fortunei* and *C. griffithii* clade, all nodes within the genus are robust with a BS and PP of 100 % and 1.0, respectively (Fig. 4).

### Divergence time estimation

**Fig. 5.**
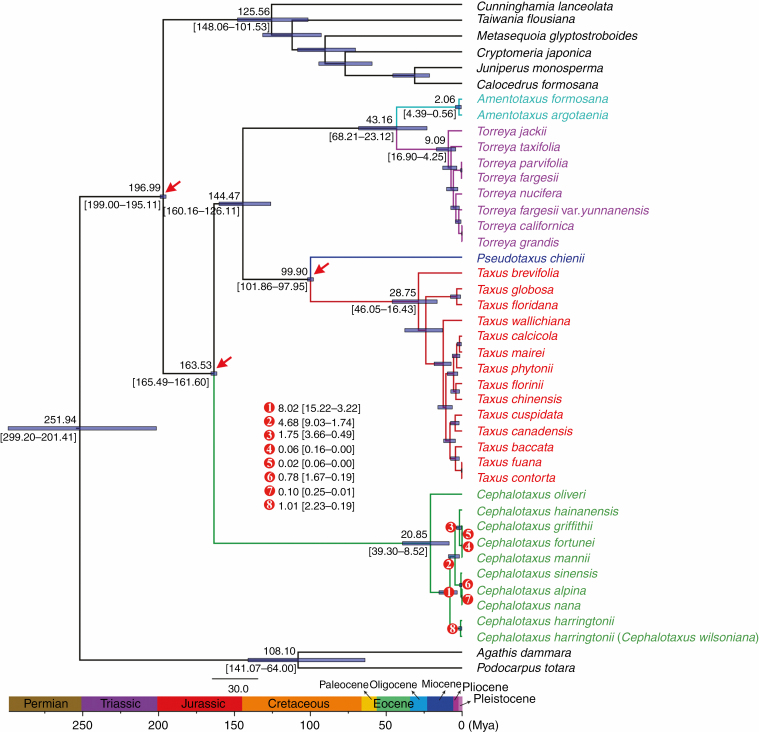
Divergence time estimation based on 81 plastid protein-coding genes. Numbers above and under the branches represent mean divergent ages and 95 % confidence interval of each node, respectively. Red arrows show the calibrating points for molecular dating. Divergence time and the timeline are indicated in million years ago (Mya).

The fossil-calibrated molecular dating (Fig. 5) indicated that the diversification of extant *Cephalotaxus* species initiated around 20.85 Mya [95 % highest posterior density (HPD): 39.30–8.52 Mya], which is the Oligocene/Miocene transition, with *C. oliveri* being the earliest diverging species in the genus. Subsequently, the speciation of *C. harringtonii* occurred at 8.02 Mya (95 % HPD: 15.22–3.22 Mya), in the late Miocene. The diversification of the remaining species was dated to 4.68 Mya (95 % HPD: 9.03–3.22 Mya), around the Miocene/Pliocene boundary, leading to the split of the clade including *C. hainanensis*, *C. mannii*, *C. fortunei* and *C. griffithii*, and the other consisting of *C. sinensis*, *C. alpina* and *C. nana.* The divergence of the aforementioned terminal species occurred in the Pleistocene. Additionally, the BEAST analyses assumed a median age of 144.47 Mya (95 % HPD: 160.16–126.11 Mya) for the crown age of Taxaceae. The crown ages of *Amentotaxus*, *Torreya*, and *Taxus* were dated to 2.06 Mya (95 % HPD: 4.39–0.56 Mya), 9.09 Mya (95 % HPD: 16.90–4.25 Mya) and 28.75 Mya (95 % HPD: 46.05–16.43 Mya), respectively. Similar to *Cephalotaxus*, the most extensive species divergence events in these genera, which are responsible for the occurrence of the majority of the extant species in each genus, occurred during late Miocene to Pleistocene (Fig. 5).

## DISCUSSION

### Phylogenetic inferences and taxonomic implications

Previous studies based on single- or multilocus DNA sequence data failed to recover an unambiguous relationship between *Cephalotaxus* and closely related genera (Chaw *et al.*, 1997; Cheng *et al.*, 2000; Quinn *et al.*, 2002; Hao *et al.*, 2008; Lu *et al.*, 2014). Based on a large dataset of 81 protein-coding genes that contain more variable sites and parsimoniously informative variations than have previously been available (Supplementary data Table S2), and by far the most comprehensive taxonomic sampling (all extant *Cephalotaxus* species were included, and *Taxus* and *Torreya* were well represented), our plastome phylogenomic analyses not only reconstructed robust relationships of *Cephalotaxus* to allied genera, with high support for each intergeneric node (Fig. 4), but also provided a well-supported resolution of interspecific relationships within *Cephalotaxus*. Our results further confirmed that phylogenetic reconstruction based on plastome sequence data can effectively resolve historical problems in phylogenetically perplexing plant groups.

Although the sister relationship between *Torreya* and *Amentotaxus* has been commonly revealed by previous studies (Chaw *et al.*, 1997; Cheng *et al.*, 2000; Wang and Shu, 2000; Quinn *et al.*, 2002; Hao *et al.*, 2008; Lu *et al.*, 2014; Majeed *et al.*, 2018; Ran *et al.*, 2018; X. Zhang *et al.*, 2019), the position of these two genera remains unresolved. For instance, the phylogenetic analyses based on 18S ribosomal DNA (Chaw *et al.*, 1997), ribosomal ITS regions (Cheng *et al.*, 2000; Hao *et al.*, 2008), *LFY* and *NLY* genes (Lu *et al.*, 2014) and RNA sequencing (Majeed *et al.*, 2018) revealed that they are closer to *Cephalotaxus* (Cephalotaxaceae) than to Taxaceae. However, phylogenetic reconstruction using plastid *matK* (Cheng *et al.*, 2000), a combination of plastid *matK*, *rbcL*, *trnL–F* and *psbA–trnH* (Hao *et al.*, 2008), transcriptomic data (Ran *et al.*, 2018) and plastomes (X. Zhang *et al.*, 2019; this study) yielded opposite results. Notably, those studies that reconstructed phylogeny based on nuclear sequence data, i.e. Chaw *et al.* (1997), Cheng *et al.* (2000), Lu *et al.* (2014) and Majeed *et al.* (2018), had limited taxonomic sampling from Cephalotaxaceae and Taxaceae. Accordingly, phylogenetic errors or uncertainty in the tree topology resulting from limited taxon sampling seem inevitable (Rokas and Carroll, 2005; Philippe *et al.*, 2011). Although the ribosomal ITS sequences of a wider spectrum of taxon representing Cephalotaxaceae and Taxaceae were analysed by Hao *et al.* (2008), neither ML nor BI phylogeny was able to provide strong support to the sister relationship between *Cephalotaxus* and the *Torreya* + *Amentotaxus* clade.

With a much larger taxon sampling than has previously been available, plastome phylogenomics were capable of providing valuable and credible insights for elucidating the long-standing controversies in the relationships among *Amentotaxus*, *Cephalotaxus*, *Pseudotaxus*, *Taxus* and *Torreya*, and thus contribute to a clear-cut delineation between Cephalotaxaceae and Taxaceae. Briefly, *Cephalotaxus* (Cephalotaxaceae) was resolved as sister to Taxaceae; within Taxaceae, the sister relationships between *Taxus* and *Pseudotaxus*, as well as between *Torreya* and *Amentotaxus*, were strongly supported (Fig. 5). Although the plastomes of *Cephalotaxus* and allied genera are paternally inherited (Mogensen, 1996) and analysis of plastome DNA sequences can only represent the paternal histories of these genera, the relationships among these genera revealed by our data are highly congruent with that of transcriptome-based phylogeny (Ran *et al.*, 2018). This implies that the previous disagreements regarding the systematic position of *Amentotaxus* and *Torreya* most probaby result from limited taxonomic sampling (e.g. Chaw *et al.*, 1997; Lu *et al.*, 2014; Majeed *et al.*, 2018) or inadequate sequence variations (Hao *et al.*, 2008) rather than conflicts between plastid and nuclear datasets. In view of this, the plastome-based phylogeny reconstructed in this study can credibly reflect the evolutionary relationships among these genera.

The relationships revealed by our data are consistent with some morphological characteristics: the two-ovulate bracts in the seed cones distinguish *Cephalotaxus* from the single-ovulate bracts of the rest of the genera (Xiao *et al.*, 2008); among the Taxaceae genera, the sister relationship between *Taxus* and *Pseudotaxus* is supported by the seeds being imperfectly enveloped by a cup-like aril, in contrast to seeds completely enclosed by aril in the sister genera *Torreya* and *Amentotaxus* (Price, 1990). Remarkably, large-scale plastome structural rearrangements were observed in Cephalotaxaceae, making it distinct from Taxaceae in the organization of plastid genes (Fig. 2). In addition to morphological differences (Xiao *et al.*, 2008), embryological investigations also identified clear distinctions between Cephalotaxaceae and Taxaceae (Singh, 1961). Collectively, these differences convincingly justify the distinctiveness of Cephalotaxaceae and Taxaceae, and thus they should be recognized as separate families. Therefore, our results offer plastome-based phylogenomic evidence to recognize Cephalotaxaceae as a monotypic family sister to Taxaceae (Cheng *et al.*, 2000), and support the classification proposed by Janchen (1949), who divided Taxaceae into two tribes, Torreyeae (including *Amentotaxus* and *Torreya*) and Taxeae (including *Pseudotaxus* and *Taxus*).

The robust phylogeny reconstructed in this study also provides new insights into the species delineation in *Cephalotaxus* (Fig. 4). For instance, the well-supported sister relationship between *C. wilsoniana* and *C. harringtonii* was recovered, providing molecular evidence to support treating *C. wilsoniana* as either a conspecific variety (Fu, 1999; Farjon, 2010) or a synonym of the latter species (Lang *et al.*, 2013; J. W. Zhang *et al.*, 2019). In addition, the tree topologies indicated that *C. alpina* is phylogenetically distinct from *C. fortunei*, supporting that it should be recognized as a separate species (Fu, 1984; Lang *et al.*, 2013; J. W. Zhang *et al.*, 2019) rather than a conspecific variety under *C. fortunei* (Fu, 1999; Farjon, 2010). Nevertheless, as shown by our results, the treatment of *C. mannii* as a synonym of either *C. harringtonii* (Lang *et al.*, 2013) or *C. hainanensis* (J. W. Zhang *et al.*, 2019), as well as reducing *C. sinensis* to *C. harringtonii* (Lang *et al.*, 2013; J. W. Zhang *et al.*, 2019) are likely to be arbitrary.

Notably, none of the taxonomic revisions of *Cephalotaxus* is fully supported by our data, suggesting that the alpha taxonomy of the genus is still not well understood. Recently, DNA barcodes have been widely used for delineating species, particularly in taxonomically difficult plant groups. Because of their low effectiveness in discriminating recently diverged taxa, the standard DNA barcodes (i.e. *rbcL*, *psbA–trnH* and ITS) yield a weak performance in identifying *Cephalotaxus* species (Gao *et al.*, 2015). Our results show that analysis of the plastome DNA sequence can significantly improve phylogenetic resolution in this genus. In addition, the great potential of plastome sequencing to accurately and reliably delineate species in taxonomically challenging plant genera, such as *Araucaria* (Ruhsam *et al.*, 2015), *Dendrobium* (Zhu *et al.*, 2019), *Panax* (Ji *et al.*, 2019*b*), *Paris* (Ji *et al.*, 2020) and *Taxus* (Fu *et al.*, 2019), has been demonstrated in recent studies. Therefore, analyses of plastome DNA sequences by sampling multiple individuals from multiple congeneric species will help future taxonomic revision of *Cephalotaxus* through credible species delineation.

### *Recent species divergence in* Cephalotaxus *and allied genera*

The divergence of *Cephalotaxus* species can be attributed to climate changes in the Cenozoic, in particular the establishment of monsoonal climates in East Asia and the Pleistocene climatic fluctuations. The initiation of the Asian monsoons in the Oligocene built a connection between forests from low to high latitudes of East Asia (Sun and Wang, 2005) and a humid climate in sub-tropical areas (Wan *et al.*, 2007; Jacques *et al.*, 2011). From that period onward, the forest corridor would allow the ancestral lineage of extant *Cephalotaxus* species to retreat to sub-tropical areas in response to the Neogene global cooling (Zachos *et al*., 2001, 2008). Interestingly, the earliest occurrence of *Cephalotaxus* in sub-tropical areas is a fossil species (*C. ningmingensis*) found in the Oligocene Ningming Formation of Guangxi, South China, which is most similar to extant *C. oliveri* in morphology (Shi *et al.*, 2010). In addition, the early divergence of *Cephalotaxus*, resulting in the speciation of *C. oliveri*, was dated to the Oligocene/Miocene boundary (Fig. 5). Collectively, it is reasonable to hypothesize that the colonization of *Cephalotaxus* into low latitudes of East Asia, as well as the divergence of *C. oliveri*, could be attributed to the combination of the Neogene global climate cooling and the onset of the Asian monsoon.

The secondary divergence event in *Cephalotaxus*, involving the speciation of *C. harringtonii*, was dated at 8.02 Mya, around the late Miocene (Fig. 5). Since then, the intensification of Asian summer monsoons established a humid climate and caused a significant expansion of forests in East Asia (Sun and Wang, 2005; Yao *et al.*, 2011). Such climatic and environmental shifts would drive the divergence of *C. harringtonii* and its northward migration to Japan and the Korean Peninsula. Moreover, the Middle Miocene fossil record of *Cephalotaxus* from Yunnan, south-west China (J. W. Zhang *et al.*, 2019), suggests that the enhancement of monsoonal climates in East Asia played an important role in driving the westward dispersal of *Cephalotaxus* as well.

It is noteworthy that the most extensive species divergence events in *Cephalotaxus*, resulting in the divergence of the majority of the extant species, occurred in the Pleistocene (Fig. 5). During the Pleistocene, there were at least four major glaciations in East Asia (Shi *et al.*, 2005). The Pleistocene glaciation/interglaciation cycles, which caused dramatic contraction/expansion of species ranges in the Northern Hemisphere (Axelrod *et al.*, 1998; Qian and Ricklefs, 2000; Petit, 2003; Qiu *et al.*, 2011), plus the increased complexity of topography in East Asia which might have blocked the regional gene flow and boosted vicariance (Wen *et al.*, 2014; Favre *et al.*, 2015), are believed to have triggered rapid speciation in many plant lineages in East Asia (Li and Li, 1997; Axelrod *et al.*, 1998; Wu *et al.*, 2005; Lu *et al.*, 2018). Similarly, these events would have triggered significant species radiation in *Cephalotaxus.*

Both fossil evidence (Florin, 1958, 1963; He and Tao, 1997; Manchester *et al.*, 2009; Leslie, 2018) and molecular dating consistently indicate that *Cephalotaxus* and all Taxaceae genera are ancient plant lineages. The antiquity of these genera can also be justified by the structural divergences among their plastomes. Despite high levels of similarity in gene content, massive genic rearrangements observed at the genus level (Fig. 2) suggest that plastomes are highly divergent in gene organization. The major differences are most likely to be associated with long-term divergent evolution of plastomes among these genera and thus accumulation of mutations and structural variations as inversions and relocation of genes. Nevertheless, divergence time estimation indicates that all extant species within the genus *Cephalotaxus* derived from recent divergence events that initiated around the Oligocene/Miocene transition and intensified in the Pleistocene. The young ages of extant *Cephalotaxus* species are consistent with the low levels of DNA sequence variations among their plastomes (Fig. 3). Recent diversification of extant *Cephalotaxus* species implies that they are not evolutionary relicts.

We found a similar scenario of species diversification in *Amentotaxus*, *Taxus* and *Torreya*: most extant species of these genera also originated from recent speciation events occurring in the late Miocene, Pliocene and Pleistocene, despite the origins of these genera occurring no later than the Paleogene according to the fossil record (Florin, 1958, 1963; Manchester *et al.*, 2009; Leslie, 2018). Recent diversification of extant species has been observed in numerous ancient gymnosperm lineages. For instance, fossil-calibrated molecular phylogenies indicate that extant cycad species are not much older than approx. 12 million years although the cycad lineage is ancient (Nagalingum *et al.*, 2011). Recent radiations have also been reported in many coniferous lineages in East Asia, such as *Cupressus* (Xu *et al.*, 2010), *Juniperus* (Li *et al.*, 2012), *Larix* (Wei and Wang, 2004), *Picea* (Ran *et al*., 2006, 2015) and *Pinus* (Liu *et al.*, 2014; Hao *et al.*, 2015; Liu *et al.*, 2019). The scenario of ancient lineages possessing extremely high diversity of recently diverged species suggests that these gymnosperm genera may have suffered considerable extinction during their evolutionary histories (Nagalingum *et al.*, 2011). Therefore, occurrence of such ancient lineages in a certain area does not equate to antiquity of the extant flora, as revealed by our results and previous studies.

### Conclusions

This study is by far the most comprehensive taxonomic sampling, plastome-based phylogenomic inference of *Cephalotaxus*. The robust phylogeny reconstructed in this study provides a perspective on the phylogenetic placement of *Cephalotaxus*, and contributes to a clear-cut delineation between Cephalotaxaceae and Taxaceae. The well-supported resolution of interspecific relationships within *Cephalotaxus* provides new evidence to clarify some of the long-standing debates in species delineation. Our data reveal that the divergence of *Cephalotaxus* can be attributed to the establishment of monsoonal climates in East Asia and the Pleistocene climatic fluctuations, and the species richness in this genus is associated with recent divergence events occurring in the Pleistocene. Our findings reject the idea that extant *Cephalotaxus* species are evolutionary relicts (Fu, 1984; Wu *et al.*, 2005). The evolutionary profiles of *Cephalotaxus* provide insightful knowledge for understanding the origin and evolution of the floristic paleo-endemism in East Asia.

## SUPPLEMENTARY DATA

Supplementary data are available online at https://academic.oup.com/aob and consist of the following. Table S1: plastome sequences downloaded from GenBank. Table S2: sequence characteristics of 81 protein-coding genes involved in the phylogenetic analyses. Table S3: summary of Illumina sequencing of *Cephalotaxus* species. Table S4: comparison of plastome features among *Cephalotaxus* species. Table S5: gene content of *Cephalotaxus* plastomes.

mcaa201_suppl_Supplementary_Table_S1

mcaa201_suppl_Supplementary_Table_S2

mcaa201_suppl_Supplementary_Table_S3

mcaa201_suppl_Supplementary_Table_S4

mcaa201_suppl_Supplementary_Table_S5
