# Supplementary material for: Plastome phylogenomics of Cephalotaxus (Cephalotaxaceae) and allied genera
Source: Ann Bot. 2020 Nov 30;127(5):697–708. doi: 10.1093/aob/mcaa201 (PMC8052924; doi:10.1093/aob/mcaa201)
Supplement: mcaa201_suppl_Supplementary_Table_S1 [file mcaa201_suppl_supplementary_table_s1.doc]

Table S1. Plastome sequences downloaded from GeneBank.

| Taxon | Family | GenBank Accession |
| --- | --- | --- |
| *Cephalotaxus wilsoniana* | Cephalotaxaceae | NC_016063 |
| *Calocedrus formosana* | Cupressaceae | AB831010 |
| *Cryptomeria japonica* | Cupressaceae | AP009377 |
| *Cunninghamia lanceolata* | Cupressaceae | NC_021437 |
| *Juniperus monosperma* | Cupressaceae | KF866298 |
| *Metasequoia glyptostroboides* | Cupressaceae | KR061358 |
| *Taiwania flousiana* | Cupressaceae | KC427274 |
| *Amentotaxus argotaenia* | Taxaceae | NC_027581 |
| *Amentotaxus formosana* | Taxaceae | NC_024945 |
| *Pseudotaxus chienii* | Taxaceae | NC_041503 |
| *Taxus baccata* | Taxaceae | KR476375 |
| *Taxus brevifolia* | Taxaceae | MH390457 |
| *Taxus calcicola* | Taxaceae | MH390451 |
| *Taxus canadensis* | Taxaceae | NC_041499 |
| *Taxus chinensis* | Taxaceae | MH390476 |
| *Taxus contorta* | Taxaceae | MH390455 |
| *Taxus cuspidata* | Taxaceae | MH390447 |
| *Taxus floridana* | Taxaceae | MH390468 |
| *Taxus florinii* | Taxaceae | MH390463 |
| *Taxus fauna* | Taxaceae | MF278259 |
| *Taxus globose* | Taxaceae | MH390467 |
| *Taxus mairei* | Taxaceae | KJ123824 |
| *Taxus phytonii* | Taxaceae | MH390470 |
| *Taxus wallichiana* | Taxaceae | MF850258 |
| *Torreya californica* | Taxaceae | MK249062 |
| *Torreya fargesii* | Taxaceae | NC_029398 |
| *Torreya grandis* | Taxaceae | NC_034806 |
| *Torreya nucifera* | Taxaceae | MK978775 |
| *Torreya parvifolia* | Taxaceae | NC_043866 |
| *Torreya taxifolia* | Taxaceae | MK249063 |
| *Agathis dammara* (Outgroup) | Araucariaceae | AB830884 |
| *Podocarpus totara* (Outgroup) | Podocarpaceae | KC306742 |
