# Supplementary material for: Plastome phylogenomics of Cephalotaxus (Cephalotaxaceae) and allied genera
Source: Ann Bot. 2020 Nov 30;127(5):697–708. doi: 10.1093/aob/mcaa201 (PMC8052924; doi:10.1093/aob/mcaa201)
Supplement: mcaa201_suppl_Supplementary_Table_S2 [file mcaa201_suppl_supplementary_table_s2.docx]

Table S2. Sequence characteristics of 81 protein-encoding genes involved in the phylogenetic analyses.

| Gene | Aligned length (bp) | No. of variable sites  (divergence%) | No. of parsimony informative sites (divergence%) |
| --- | --- | --- | --- |
| *accD* | 4,463 | 2,221 (49.76) | 1,738 (38.94) |
| *atpA* | 1,506 | 319 (21.18) | 189 (12.55) |
| *atpB* | 1,548 | 335 (21.64) | 211 (13.63) |
| *atpE* | 411 | 135 (32.85) | 82 (19.95) |
| *atpF* | 552 | 178 (32.25) | 95 (17.21) |
| *atpH* | 246 | 34 (13.82) | 20 (8.13) |
| *atpI* | 750 | 181 (24.13) | 110 (14.67) |
| *ccsA* | 975 | 306 (31.38) | 193 (19.79) |
| *cemA* | 786 | 249 (31.68) | 153 (19.47) |
| *chlB* | 1,539 | 384 (24.95) | 216 (14.04) |
| *chlL* | 885 | 163 (18.42) | 106 (11.98) |
| *chlN* | 1,377 | 328 (23.82) | 195 (14.16) |
| *clpP* | 612 | 423 (69.12) | 360 (58.82) |
| *infA* | 211 | 135 (63.98) | 122 (57.82) |
| *matK* | 1,509 | 621 (41.15) | 372 (24.65) |
| *ndhA* | 1,101 | 229 (20.80) | 128 (11.63) |
| *ndhB* | 1,176 | 308 (26.19) | 182 (15.48) |
| *ndhC* | 363 | 89 (24.52) | 52 (14.33) |
| *ndhD* | 1,500 | 369 (24.60) | 226 (15.07) |
| *ndhE* | 303 | 54 (17.82) | 29 (9.57) |
| *ndhF* | 2,260 | 665 (29.42) | 429 (18.98) |
| *ndhG* | 546 | 130 (23.81) | 90 (16.48) |
| *ndhH* | 1,176 | 200 (17.01) | 114 (9.69) |
| *ndhI* | 482 | 99 (20.54) | 61 (12.66) |
| *ndhJ* | 477 | 110 (23.06) | 75 (15.72) |
| *ndhK* | 764 | 232 (30.37) | 150 (19.63) |
| *petA* | 966 | 226 (23.40) | 148 (15.32) |
| *petB* | 648 | 115 (17.75) | 72 (11.11) |
| *petD* | 426 | 72 (16.90) | 49 (11.50) |
| *petG* | 114 | 25 (21.93) | 14 (12.28) |
| *petL* | 96 | 34 (35.42) | 24 (25.00) |
| *petN* | 90 | 12 (13.33) | 8 (8.89) |
| *psaA* | 2,253 | 364 (16.16) | 235 (10.43) |
| *psaB* | 2,205 | 365 (16.55) | 228 (10.34) |
| *psaC* | 246 | 26 (10.57) | 16 (6.50) |
| *psaI* | 111 | 47 (42.34) | 25 (22.52) |
| *psaJ* | 129 | 44 (34.11) | 23 (17.83) |
| *psaM* | 93 | 34 (36.56) | 14 (15.05) |
| *psbA* | 1,062 | 124 (11.68) | 68 (6.40) |
| *psbB* | 1,527 | 250 (16.37) | 171 (11.20) |
| *psbC* | 1,422 | 245 (17.23) | 152 (10.69) |
| *psbD* | 1,062 | 121 (11.39) | 75 (7.06) |
| *psbE* | 258 | 42 (16.28) | 23 (8.91) |
| *psbF* | 120 | 19 (15.83) | 10 (8.33) |
| *psbH* | 228 | 56 (24.56) | 41 (17.98) |
| *psbI* | 108 | 24 (22.22) | 14 (12.96) |
| *psbJ* | 123 | 44 (35.77) | 25 (20.33) |
| *psbK* | 189 | 49 (25.93) | 31 (16.40) |
| *psbL* | 117 | 18 (15.38) | 8 (6.84) |
| *psbM* | 105 | 41 (39.05) | 29 (27.62) |
| *psbN* | 132 | 22 (16.67) | 11 (8.33) |
| *psbT* | 108 | 19 (17.59) | 13 (12.04) |
| *psbZ* | 189 | 37 (19.58) | 23 (12.17) |
| *rbcL* | 1,452 | 287 (19.77) | 165 (11.36) |
| *rpl2* | 843 | 246 (29.18) | 172 (20.40) |
| *rpl14* | 369 | 92 (24.93) | 68 (18.43) |
| *rpl16* | 375 | 100 (26.67) | 82 (21.87) |
| *rpl20* | 369 | 121 (32.79) | 87 (23.58) |
| *rpl22* | 336 | 171 (50.89) | 139 (41.37) |
| *rpl23* | 288 | 102 (35.42) | 80 (27.78) |
| *rpl32* | 88 | 39 (44.32) | 30 (34.09) |
| *rpl33* | 198 | 66 (33.33) | 39 (19.70) |
| *rpl36* | 114 | 36 (31.58) | 29 (25.44) |
| *rpoA* | 1,035 | 386 (37.29) | 251 (24.25) |
| *rpoB* | 3,459 | 1,191 (34.43) | 758 (21.91) |
| *rpoC1* | 2,721 | 866 (31.83) | 548 (20.14) |
| *rpoC2* | 3,866 | 1,386 (35.85) | 884 (22.87) |
| *rps2* | 696 | 244 (35.06) | 174 (25.00) |
| *rps3* | 765 | 290 (37.91) | 212 (27.71) |
| *rps4* | 723 | 260 (35.96) | 166 (22.96) |
| *rps7* | 396 | 137 (34.60) | 98 (24.75) |
| *rps8* | 348 | 117 (33.62) | 76 (21.84) |
| *rps11* | 370 | 135 (36.49) | 101 (27.30) |
| *rps14* | 303 | 78 (25.74) | 57 (18.81) |
| *rps15* | 267 | 98 (36.70) | 69 (25.84) |
| *rps18* | 237 | 97 (40.93) | 78 (32.91) |
| *rps19* | 288 | 103 (35.76) | 75 (26.04) |
| *ycf1* | 10,897 | 5,963 (54.72) | 4,400 (40.38) |
| *ycf2* | 11,369 | 5,081 (44.69) | 3,499 (30.78) |
| *ycf3* | 528 | 79 (14.96) | 48 (9.09) |
| *ycf4* | 555 | 143 (25.77) | 90 (16.22) |
