# Supplementary material for: Plastome phylogenomics of Cephalotaxus (Cephalotaxaceae) and allied genera
Source: Ann Bot. 2020 Nov 30;127(5):697–708. doi: 10.1093/aob/mcaa201 (PMC8052924; doi:10.1093/aob/mcaa201)
Supplement: mcaa201_suppl_Supplementary_Table_S3 [file mcaa201_suppl_supplementary_table_s3.doc]

Table S3. Summary of Illumina sequencing of *Cephalotaxus* species.

| Species | Voucher | No. of clean reads | Plastid-like reads | Sequencing coverage (X) |
| --- | --- | --- | --- | --- |
| *Cephalotaxus n*a*na* | JYH2017090 | 30,108,972 | 485,042 | 541.595 |
| [*C. griffithii*](https://en.wikipedia.org/wiki/Cephalotaxus_griffithii) | JYH2017095 | 30,166,636 | 819,866 | 915.458 |
| [*C. fortunei*](https://en.wikipedia.org/wiki/Cephalotaxus_fortunei) | JYH2017084 | 30,328,830 | 452,272 | 505.005 |
| [*C. mannii*](https://en.wikipedia.org/wiki/Cephalotaxus_mannii) | JYH2017118 | 30,889,644 | 737,173 | 823.124 |
| [*C. hainanensis*](https://en.wikipedia.org/wiki/Cephalotaxus_hainanensis) | JYH2017124 | 30,918,904 | 625,915 | 698.893 |
| *C. alpina* | JYH2017120 | 31,168,262 | 624,497 | 697.310 |
| [*C. sinensis*](https://en.wikipedia.org/wiki/Cephalotaxus_sinensis) | JYH2017094 | 31,372,628 | 1,000,410 | 1117.053 |
| [*C. oliveri*](https://en.wikipedia.org/wiki/Cephalotaxus_oliveri) | JYH2017121 | 31,432,028 | 877,768 | 980.111 |
| [*C. harringtonii*](https://en.wikipedia.org/wiki/Cephalotaxus_harringtonii) | JYH2017119 | 31,543,304 | 416,005 | 464.509 |
