# Supplementary material for: Plastome phylogenomics of Cephalotaxus (Cephalotaxaceae) and allied genera
Source: Ann Bot. 2020 Nov 30;127(5):697–708. doi: 10.1093/aob/mcaa201 (PMC8052924; doi:10.1093/aob/mcaa201)
Supplement: mcaa201_suppl_Supplementary_Table_S4 [file mcaa201_suppl_supplementary_table_s4.doc]

*Table S4. Comparison of plastome features among Cephalotaxus species.*

| **Species** | **Total** | |  | **Coding regions** | |  | **Noncoding regions** | |
| --- | --- | --- | --- | --- | --- | --- | --- | --- |
|  | **length** | **GC content (%)** |  | **length** | **%GC** |  | **length** | **%GC** |
| *Cephalotaxus alpina* | 136,368 | 35.1 |  | 83,841 | 37.4 |  | 52,527 | 31.5 |
| *C. fortunei* | 136,195 | 35.1 |  | 83,820 | 37.4 |  | 52,375 | 31.5 |
| *C. griffithii* | 135,778 | 35.1 |  | 83,481 | 37.4 |  | 52,297 | 31.5 |
| *C. hainanensis* | 136,545 | 35.1 |  | 83,838 | 37.3 |  | 52,707 | 31.4 |
| *C. harringtonii* | 135,896 | 35.1 |  | 83,593 | 37.4 |  | 52,303 | 31.5 |
| *C. harringtonii* (*C. wilsoniana*) | 136,196 | 35.1 |  | 83,919 | 37.3 |  | 52,277 | 31.5 |
| *C. mannii* | 136,528 | 35.1 |  | 83,985 | 37.3 |  | 52,543 | 31.6 |
| *C. nana* | 135,887 | 35.1 |  | 83,427 | 37.4 |  | 52,460 | 31.5 |
| *C. oliveri* | 134,550 | 35.2 |  | 82,928 | 37.5 |  | 51,622 | 31.6 |
| *C. sinensis* | 136,434 | 35.1 |  | 83,883 | 37.4 |  | 52,551 | 31.5 |
