# Supplementary material for: Plastome phylogenomics of Cephalotaxus (Cephalotaxaceae) and allied genera
Source: Ann Bot. 2020 Nov 30;127(5):697–708. doi: 10.1093/aob/mcaa201 (PMC8052924; doi:10.1093/aob/mcaa201)
Supplement: mcaa201_suppl_Supplementary_Table_S5 [file mcaa201_suppl_supplementary_table_s5.doc]

Table S5. Gene content of *Cephalotaxus* plastomes.

| Category of Genes | Gene group | Gene name |
| --- | --- | --- |
| Self-replication | Ribosomal RNA genes | *rrn*4.5, *rrn*5, *rrn*16, *rrn*23 |
|  | Transfer RNA genes | *trn*A-UGC*, *trn*C-GCA, *trn*D-GUC, *trn*E-UUC, *trn*F-GAA, *trn*fM-CAU, *trn*G-GCC, *trn*G-UCC*, *trn*H-GUG, *trn*I-CAU, *trn*I-GAU*, *trn*K-UUU*, *trn*L-CAA, *trn*L-UAG, *trn*M-CAU, *trn*N-GUU, *trn*P-GGG, *trn*P-UGG, *trn*Q-UUG×2, *trn*R-ACG, *trn*R-UCU, *trn*S-GCU, *trn*S-GGA, *trn*S-UGA, *trn*T-GGU, *trn*W-CCA, *trn*Y-GUA |
|  | Ribosomal protein  (small subunit) | *rps*2, *rps*3, *rps*4, *rps*7, *rps*8, *rps*11, *rps*12**, *rps*14, *rps*15, *rps*16*, *rps*18, *rps*19 |
|  | Ribosomal protein  (large subunit) | *rpl*2*, *rpl*14, *rpl*16*, *rpl*20, *rpl*22, *rpl*23, *rpl*32, *rpl*33, *rpl*36 |
|  | RNA polymerase | *rpo*A, *rpo*B, *rpo*C1*, *rpo*C2 |
|  | Translational initiation factor | *inf*A |
| Genes for photosynthesis | Subunits of photosystem I | *psa*A, *psa*B, *psa*C, *psa*I, *psa*J, *psa*M, *ycf3***, *ycf*4 |
|  | Subunits of photosystem II | *psb*A, *psb*B, *psb*C, *psb*D, *psb*E, *psb*F, *psb*H, *psb*I, *psb*J, *psb*K, *psb*L, *psb*M, *psb*N, *psb*T, *psb*Z |
|  | Subunits of cytochrome | *pet*A, *pet*B*, *pet*D*, *pet*G, *pet*L, *pet*N |
|  | Subunits of ATP synthase | *atp*A, *atp*B, *atp*E, *atp*F*, *atp*H, *atp*I |
|  | Large subunit of Rubisco | *rbc*L |
|  | Subunits of NADH  dehydrogenase | *ndh*A*, *ndh*B*, *ndh*C, *ndh*D, *ndh*E, *ndh*F, *ndh*G, *ndh*H, *ndh*I, *ndh*J, *ndh*K, |
|  | Chlorophyll biosynthesis | *chl*B, *chI*L, *chI*N |
| Other genes | Maturase | *mat*K |
|  | Envelope membrane protein | *cem*A, |
|  | Subunit of acetyl-CoA | *acc*D |
|  | Synthesis gene | *ccs*A, |
|  | ATP-dependent protease | *clp*P |
|  | Component of TIC complex | *ycf*1 |
| Genes of  unknown function | Conserved open reading frames | *ycf*2 |

×2: Two gene copies in the IR regions; * with one intron; ** with two introns.
